# Supplementary material for: Integrated 3d flow-based multi-atlas brain structure segmentation
Source: PLoS One. 2022 Aug 15;17(8):e0270339. doi: 10.1371/journal.pone.0270339 (PMC9377636; doi:10.1371/journal.pone.0270339)
Supplement: Attachment — Submitted filename: ResponsetoReviewers.pdf [file pone.0270339.s001.pdf]

Integrated 3d flow-based multi-atlas brain structure segmentation

Authors' Response

Yeshu Li Ziming Qiu Xingyu Fan Xianglong Liu
Eric I-Chao Chang Yan Xu

We thank the editor and reviewers for the constructive comments and the general appreciation of our work. We have revised our manuscript (manuscript number: PONE-D-21-07784) accordingly. Please see below our point-by-point responses to the specific questions.

1 Response to Editor #1's Comments

We thank the editor for providing detailed comments on formatting.

Question 1: "Please ensure that your manuscript meets PLOS ONE's style requirements, including those for file naming. The PLOS ONE style templates can be found at `https://journals.plos.org/plosone/s/file?id=wjVg/PLOSOne_formatting_sample_main_body.pdf` and `https://journals.plos.org/plosone/s/file?id=ba62/PLOSOne_formatting_sample_title_authors_affiliations.pdf`"
Answer: *We adopt the official LaTeX template provided by PLOS ONE at* `https://journals.plos.org/plosone/s/file?id=SyJ3/plos-latex-template.zip` *to format our manuscript.*

Question 2: "Please include both an updated Funding Statement and Competing Interests Statement in your cover letter."
Answer: *We have included the required information in our cover letter.*

2 Response to Reviewer #1's Comments

We thank the reviewer for providing many constructive comments.

Question 1: "The SIFT flow works well on the 3D liver image segmentation, however, the brain is different from the liver in its convoluted structures. Aligning across different brain is much more challenging since different subjects may have substantial different cortex in folding, which means the alignment can cause large local deformations. This paper introduces the vector flow to do the alignment, but the smooth term in equation (3) seems not to be enough to guarantee the deformation to be smooth."
Answer: *The MRF formulation aims at minimizing the sum of three terms. In addition to the adopted truncated L1 norm thresholding to combat flow discontinuity, the small displacement term combined with the coarse-to-fine searching strategy ensures that the computed flow changes smoothly from the coarsest level to the original resolution.*

Please refer to ***Page 11, Line 239-241*** *in the manuscript for a detailed description:*

"Note that minimization of the weighted sum of the displacement term and the smoothness term ensures that the flow vector does not deviate from the zero vector drastically."

Question 2: "To solve the optimization of the 3D vector flow, the paper uses a coarse-to-fine strategy to get the optimal solution. However, in the label fusion stage, the optimization seems similar, is it beneficial to use the coarse-to-fine strategy?"
Answer: *We do adopt the coarse-to-fine strategy in message passing for label fusion. The benefit is mostly concerned with computational efficiency.*

Please refer to ***Page 18, Line 395-397*** *in the manuscript for a detailed description:*

"Similar to the previous registration method, we adopt a simpler sequential belief propagation algorithm to optimize the energy function for faster convergence, in which a coarse-to-fine propagation algorithm is adopted as well."

Question 3: "Is there any specific reason to choose grayscale value as additional features? It is interesting to know whether introducing it is beneficial to the segmentation performance. Also, is ζ a weight factor for gray scale feature? It is not clear how coefficient ζ works in the feature concatenation."
Answer: *Grayscale is the original feature for an image while SIFT is an handcrafted feature that requires non-trivial extraction. Intuitively, these two features have little mutual information thus form a richer representation than individuals. Moreover, according to some toy experiments we conduct, neither grayscale nor SIFT feature alone leads to good segmentation performance, while simple concatenation of them does. The coefficient ζ is introduced and should be tuned because the two features may have different scales. Thus it is not appropriate to simply add them up without weighting.*

Please refer to ***Page 10, Line 200-201, 206-208*** *in the manuscript for a detailed description:*

"By experiments, we find that SIFT feature and intensity include complementary information for voxel-wise alignment."

"The introduction of ζ results from the fact that neither SIFT nor grayscale alone can capture the essential information for a voxel."

Question 4: "From the paper, the author mentions their registration outperforms the ANTs. Since ANTs is a relatively old registration method, latest registration like VoxelMorph seems outperforms the ANTs, can the author discuss the use of the deep learning- based registration in the proposed framework?"
Answer: *We have made a discussion on possible use of a registration neural network in the proposed framework.*

Please refer to ***Page 35, Line 828-834*** *in the manuscript for a detailed description:*

"Deep learning models are promising and widely adopted non-linear hypothesis sets for statistical learning. Without sophisticated fine-tuning or a large hypothesis space, deep learning approaches are still not easily comparable to ANTs SyN. The models usually require a long GPU time to be trained. Notwithstanding, it would be interesting to adopt a well-trained deep learning-based registration network as our registration component. If training time is not counted in registration time, its fast inference makes a significant difference."

Question 5: "Since the end-to-end segmentation using deep learning has achieved very good performance in the segmentation, the latest segmentation network like U-net and Dense-net also achieves very good performance in the brain ROI segmentation, at least, the author needs to discuss the comparison with deep learning-based methods. In the paper, the author mentioned the major limitation for the deep learning method is the need of manual segmentation and computation cost, but for the patch based deep learning method, the require of the manual segmentation can be reduced. Also, the paper mainly discussed the computation cost in CPU, while use GPU is very common in many applications. Would there be any limitation of using GPU?"
Answer: *Most existing deep learning approaches do not make a relatively fair comparison to traditional multi-atlas segmentation methods. For example, in MICCAI 2012 MALC, no existing results based on deep learning have been reported to outperform JLF because the training data is very limited (15 images) for neural network models. To our understanding, GPU is still a more expensive resource than CPU and it is fair to convert GPU time to CPU time to make comparisons.*

Please refer to ***Page 35, Line 834-840*** *in the manuscript for a detailed description:*

"In datasets with limited training data, for example, MICCAI 2012, deep learning approaches are still not comparable to tradition methods such as ANTs

SyN. However, since neural network models form a larger hypothesis and are able to learn task-specific representations automatically, we expect deep learning-based segmentation methods will benefit our multi-atlas segmentation framework or outperform it completely in the form of end-to-end direct segmentation even in the scarce-data setting."

Question 6: "Minor comments, it is very strange the figures are separated with the figure captions."
Answer: *We agree that it is strange but we have double checked the format guideline of PLOS ONE and found that this is exactly a requirement of the journal.*

Please refer to the author guideline of PLOS ONE in the manuscript for a detailed description.

3 Response to Reviewer #2's Comments

We thank the reviewer for providing many constructive comments.

Question 1: "The paper is written in a good manner. Some minor touches can improve this paper more."
Answer: *Thanks for pointing them out.*

Question 2: "The quality of the figures can be improved more."
Answer: *We have made sure that the best resolution of each figure is achieved.*

Question 3: "The contributions of the authors are not clear. They have mentioned in first contribution."
Answer: *We have resummarized our contributions.*

Please refer to ***Page 5, 6, Line 111-125*** *in the manuscript for a detailed description:*

"

1. *A novel multi-atlas segmentation system based on 3D integrated flow is proposed. The integrated flow is the essential element that connects all the components in the system.*
2. *We adopt an atlas selection method based on the final convergence value of the energy function in registration. This approach estimates the best candidate atlases for fusion, which costs almost no time because it only involves trivial sorting according to the byproduct numerical values from registration.*

3. *A coarse-to-fine flow matching approach and a 3D sequential belief propagation method with improved message passing scheme are developed in registration, with a simpler sequential belief propagation method adopted in label fusion. These strategies not only make the system efficient but also lead to better segmentation results. Extensive experiments are performed on five publicly available datasets in different practical settings. Our proposed method outperforms the competitive baseline methods in both efficiency and effectiveness in our experimental settings.*

"

Question 4: "Future research directions section is core, however, it is not good at all."
Answer: *More discussions on future directions have been added.*

Please refer to ***Page 35, Line 828-840*** *in the manuscript for a detailed description:*

"Deep learning models are promising and widely adopted non-linear hypothesis sets for statistical learning. Without sophisticated fine-tuning or a large hypothesis space, deep learning approaches are still not easily comparable to ANTs SyN. The models usually require a long GPU time to be trained. Notwithstanding, it would be interesting to adopt a well-trained deep learning-based registration network as our registration component. If training time is not counted in registration time, its fast inference makes a significant difference. In datasets with limited training data, for example, MICCAI 2012, deep learning approaches are still not comparable to tradition methods such as ANTs SyN. However, since neural network models form a larger hypothesis and are able to learn task-specific representations automatically, we expect deep learning-based segmentation methods will benefit our multi-atlas segmentation framework or outperform it completely in the form of end-to-end direct segmentation even in the scarce-data setting."

Question 5: "What are the computational resources reported in the state of the art for the same purpose? - Please cite each equation and clearly explain its terms. - Clearly highlight the terms used in the algorithm and explain them in the text."
Answer: *We have made a better explanation in the Implementation Details subsection.*

Question 6: "What are the evaluations used for the verification of results?"
Answer: *Dice coefficient, Hausdorff distance and Cohen's kappa coefficient.*

Question 7: "Several paragraphs contain trivial information and should be dropped."
Answer: *Thanks for pointing it out. Change has been made accordingly.*

Question 8: "I found some English mistakes please check them."
Answer: *Grammatical issues have been double checked.*

Question 9: "Kindly refer the below paper: 1. Rajput, D.S., Basha, S.M., Xin, Q. et al. Providing diagnosis on diabetes using cloud computing environment to the people living in rural areas of India. J Ambient Intell Human Comput (2021). https://doi.org/10.1007/s12652-021-03154-4"
Answer: *We have cited this paper [1].*

Please refer to ***Page 34, Line 812-814*** *in the manuscript for a detailed description:*

"In our experiments, the obviously separable registration tasks are distributed to cluster computing [1] nodes, which indeed reduces considerable time for a complete experiment from about several months to several hours."

References

[1] Dharmendra Singh Rajput, Syed Muzamil Basha, Qin Xin, Thippa Reddy Gadekallu, Rajesh Kaluri, Kuruva Lakshmanna, and Praveen Kumar Reddy Maddikunta. Providing diagnosis on diabetes using cloud computing environment to the people living in rural areas of india. *Journal of Ambient Intelligence and Humanized Computing*, pages 1–12, 2021.
