# Supplementary material for: Integrated 3d flow-based multi-atlas brain structure segmentation
Source: PLoS One. 2022 Aug 15;17(8):e0270339. doi: 10.1371/journal.pone.0270339 (PMC9377636; doi:10.1371/journal.pone.0270339)
Supplement: Attachment — Submitted filename: IntFlow_PONE_Rebuttal_1.pdf [file pone.0270339.s002.pdf]

Integrated 3d flow-based multi-atlas brain structure segmentation

Authors' Response

Yeshu Li Ziming Qiu Xingyu Fan Xianglong Liu
Eric I-Chao Chang Yan Xu

We thank the editor and reviewers for the constructive comments and the general appreciation of our work. We have revised our manuscript (manuscript number: PONE-D-21-07784R1) accordingly. Please see below our point-by-point responses to the specific questions.

1 Response to Reviewer #1's Comments

We thank the reviewer for the inspiring comments.

Question 1: "For the smoothness issue, the reviewer means for neighboring voxels, their displacement may have relatively large differences. This can be easily inferred from eq. (3), for the voxels with larger displacement field, the neighborhood displacement would have no effect, because it will automatically choose the lower bound, i.e., d. This means when doing the optimization, the neighborhood regularization term may not contribute to the objective function if the previous L1 norm cannot limit the displacement vector to be sufficient small. For the brain registrations, it is agreed for some region, the displacement can be large due to the substantial structure difference across individuals. At those regions, would the neighborhood smoothness term fail and cause the displacement field non-smooth? It is better to show some displacement field visualization figures to illustrate this?"
Answer: *Preserving discontinuity is actually desirable in applications like brain image registration because even in homogeneous regions where intensities are similar, it is still possible for them to belong to different anatomical areas defined by experts. We have added a figure to compare a discontinued deformation field, a smoother filed and a very smooth field produced by ANTs.*

Please refer to ***Line 248-255, Figure 4*** *in the manuscript for a detailed description.*

Question 2: "For the comparison to the deep learning-based method, is there any reference or experiments support the expression "In datasets with limited training data, for example, MICCAI 2012, deep learning approaches are still not comparable to traditional methods such as ANTs SyN." in the paper. The current clarification seems not convincing."
Answer: *Thank you for pointing it out. We have added references to consolidate our arguments. Actually, deep learning approaches are known to be data-hungry and are prone to overfitting especially in high-dimensional settings (few training data).*

*Please refer to **Line 884-889** in the manuscript for a detailed description.*

2 Response to Reviewer #3's Comments

We thank the reviewer for providing many constructive comments. The line numbers provided are really helpful for locating.

Question 1: "Concerning eq. 1 the authors determine the magnitude and two orientations for the gradient in 3D. The magnitude is clearly defined. For the orientations two orientations are defined, theta and phi. The authors should clearly explain, perhaps using a figure, the exact meaning of the two orientations. Are they those of spherical coordinates?"
Answer: *As the reviewer correctly points it out, the three quantities indeed form the spherical coordinate of a vector of the finite differences of a voxel. Detailed explanations have been added.*

*Please refer to **Line 177-181** in the manuscript for a detailed description.*

Question 2: "In the paragraph after eq. 1 (lines 180-185), a neighborhood of a voxel is defined by an 8x8x8 cube. Then the authors said that the concerning voxel is in the center. How is this possible if the size of the neighborhood is even (8x8x8)? Why not to have an odd number for the neighborhood size, such as 9? In this case the neighborhood will be of size 9x9x9, being the center voxel clearly defined. Using an odd number for the size, how are the sub-blocks defined?"
Answer: *In the even case, the center voxel can be any voxel in the 2x2x2 cube in the center, but the convention of choosing this position has to be consistent when computing features for all the voxels. In the odd case, the center voxel is unique, one way is to divide a 9x9x9 cube into 4 4x4x4 cubes where the voxels along the center lines are ignored. We choose an even number and a specific number by following convention in computing SIFT features and considering memory that will be occupied by the features.*

*Please refer to **Line 183-187** in the manuscript for a detailed description.*

Question 3: "A gaussian weighting function is applied for the sub-block voxels (lines 188-). This could be done in several ways. Pleas add an equation for that part. A gaussian function has the sigma parameter to be adjusted. How is

this selected? Please add a table with the 6 histogram bins, giving the specific theta and phi intervals (the authors just give a particular case in line 191). I do not understand the statement: "Such division is not disjoint thus introducing overlap among bins because this is not the geographical way of creating parallels and meridians". Same for "o overcome rotation dependence, we subtract the dominant orientation of the center voxel from all the orientations so that the dominant one points to ($\theta = 0$, $\phi = 0$) and rotation invariance is thus guaranteed." Please rewrite those expressions with some more details."

Answer: *We have added a table for that filter and revised those expressions to make SIFT computation more accessible for readers unfamiliar with the concept.*

*Please refer to **Line 192-205, Table 1** in the manuscript for a detailed description.*

Question 4: "Caption of figure 3 is talking about left-right, but figure 3 has two diagrams in the bottom-top position."

Answer: *PLOS ONE automatically flipped our figure while generating the submission file. The orientation is still correctly shown to be from left to right if the .eps file is downloaded and viewed alone. Anyway, we have rephrased the caption to avoid such confusion.*

*Please refer to **Figure 3** in the manuscript for a detailed description.*

Question 5: "Concerning eq. 3, please elaborate a bit more on the need of t and d parameters. Should they be large? Why are they needed? Comparing this eq. with eq. 6, and for the sake of homogeneity, authors could add the "+ log Z" and the end of eq. 3 (as done in eq. 6). Indeed, in the MRF context, this Z is normally referred to as "partition function". A MRF is indeed a probability field which is easy to determine out of the Energy (the E function as defined for instance in eq. 3 or the F function as defined in eq. 6). For the sake of completeness, could the authors elaborate a little bit on that? For instance, adding some equations concerning the probabilities. This way is easy to see that the achieved global minimum energy corresponds to the global maximum of the probabilistic field, i.e., the MAP solution."

Answer: *Thank you for pointing it out. We have elaborated on this point from the point of view of MAP inference in graphical models.*

*Please refer to **Line 240-243, Equation 3** in the manuscript for a detailed description.*

Question 6: "In line 262, please remove the "1" at the end of the author's name of [79]: "Shekhovtsov1" -¿ "Shekhovtsov"."

Answer: *We have removed this typo of the referenced author's name.*

Question 7: "Is possible to add a reference for paragraph in lines 305-308? In particular, the paper reads: "Hence it is not feasible in practice, especially in 3D medical image analysis." Add a reference for this or an explanation with

some evidence for that fact."

Answer: *With a time complexity of $O(n^6)$, even if n is as small as 100, this implies at least $C10^12$ arithmetic operations have to be performed for some positive integer C, which is obvious not practical because 10^8 arithmetic operations roughly require 1 second for a modern CPU to execute.*

*Please refer to **Line 322-326** in the manuscript for a detailed description.*

Question 8: "In paragraph from line 309-, the original image is downsample several times. It is well known that downsampling without pre-filtering causes aliasing. Is that fact taken into account when downsampling the images? The correct way to downsample for a 2 factor is to filter the input using a low-pass filter with half the original bandwidth and then perform the downsample (for instance keeping one sample each two). Please, elaborate a bit more on this on the paper."

Answer: *We do apply smoothing before downsampling the images in belief propagation. This point has been elaborated a bit more in the paper.*

*Please refer to **Line 351-356** in the manuscript for a detailed description.*

Question 9: "In lines 363-, the label transfer method is explained. The notation is not consistent with respect to that used in algorithm 1 (page 8 of the manuscript). In particular, in algorithm 1, atlas labels are denoted by L and L_i, and target labels with L_target, however, in lines 363- (page 16), the notation is different, c and c_i are used for the labels: c_i for the atlas and c for the target. Is possible, for instance, to used L_i and L_target?"

Answer: *We have made the notations consistent.*

*Please refer to **Line 395-398, Equation 6-7** in the manuscript for a detailed description.*

Question 10: "In lines 397-400, the manuscript reads: "It is noteworthy that in comparison to applications in natural scene images, medical image registration and label transfer applications require the coefficients of the smoothness and spatial term to be much higher because of the intensity characteristics in medical images." Please add a reference to the literature or some more evidence for this to happen. How are the "intensity characteristics in medical images"? Is that due to the fact that in medical images, the shapes are smoother (rounded)?"

Answer: *Thank you for pointing it out. We observed that the optimal parameters for smoothness terms we adopt for medical images are generally higher than that in the label transfer work [1] for natural images. We realize that this should be dependent on how the gray-scale and RGB values are normalized. So we have removed this statement.*

Question 11: "In line 402, what is the meaning for the "D" parameter? Are you considering color images or so? What is the D value for the images used in the experiments? Is it possible to consider volumes with different dimensions? Authors said NxNxN, but this is not always true: usually the volume is

NxNxM, MxNxN or NxMxN, depending of the acquisition plane (axial, sagittal, coronal)."
Answer: *$D = 3$ in our case to indicate volumetric data. We adopt the cubic size, NxNxN, for easier illustrations and experiments. For worst-case complexity analysis, we can simply take N to be the maximum of all the dimension sizes. However, our implementation works for registration between two volumes of arbitrary sizes, e.g., MxNxO and PxQxR.*

Please refer to ***Line 424*** *in the manuscript for a detailed description.*

Question 12: "In caption of table 1, please add the meaning of the following acronyms: AC, MCI, NC and LMCI. I think they are defined in the main text, but for easy reading, I think they should be defined in the caption as well."
Answer: *We have added explanations according to this good suggestion.*

Please refer to ***Table 2*** *in the manuscript for a detailed description.*

Question 13: "Concerning fig.4, is possible to merge last two rows (on the left): the two ROIs (left and right) as in the other rows? In this figure, row 4 (LPBA40), column 1, I cannot see anything in the right ROI."
Answer: *We have revised the slice screenshots to enhance their contrast and merged the two slices for EADC.*

Please refer to ***Figure 5*** *in the manuscript for a detailed description.*

Question 14: "In lines 558-559: "In integrated 3D flow-based registration, t is computed based on the current data histogram normalization thus is data-dependent", please explain. How is that performed? Which value/s is/are used for the experiments?"
Answer: *We actually use $t = \infty$ throughout the experiments because, with good preprocessed datasets, imposing a threshold on the data terms does not bring any advantages. We have revised the statement accordingly.*

Please refer to ***Line 581-582*** *in the manuscript for a detailed description.*

Question 15: "I think the acronym BP-S is not defined. Please define it first time."
Answer: *BP-S is defined earlier when we are introducing belief propagation.*

Please refer to ***Line 263*** *in the manuscript for a detailed description.*

Question 16: "In lines 563 and 564, indicate that the given value refers to the K parameter."
Answer: *Good suggestion. We have indicated that this quantity is referred to as K.*

Please refer to ***Line 584-586*** *in the manuscript for a detailed description.*

Question 17: "In lines 581 and 582, AVD is defined as Average Hausdorff Distance, but it seems more natural to use AHD as the acronym, why AVD? In addition to that, it happens that in eq. 11 a maximum operator is used to mix up the two possible Hausdorff distances. I do not understand why to say

"average" as a maximum is used. Should you change eq. 1 using a semi-sum?"
Answer: *We agree that it is more natural to use the abbreviation AHD, but we faithfully follow the convention in [2] to use AVD instead. The reference has been added here. The averaging refers to the fact we are computing the average minimum distance, inside the maximum operator, between a voxel in a volume to any voxel in the other volume. The maximum operator is adopted since the average distance from volume A to volume B has a direction, thus asymmetric. Taking the maximum makes sure that a relatively small volume contained in another volume won't make the AVD value zero out.*

Please refer to ***Line 603-604*** *in the manuscript for a detailed description.*

Question 18: "In light of eq. 12, please add the following definitions (if true): Pa=fa/N and Pc=fc/N."
Answer: *Good suggestion. The equations have been added.*

Please refer to ***Equation 12*** *in the manuscript for a detailed description.*

Question 19: "Line 611 and table 2: please make it clear that the execution time is for each of the test cases, not for whole test set. This point does not seem clear."
Answer: *We have made it more explicit that the execution time refers to the time of segmenting one volume.*

Please refer to ***Line 633-634, Table 3*** *in the manuscript for a detailed description.*

Question 20: "Fig. 5 and its caption: column (d) is for Learning to Rank atlas selection, but which registration algorithm is used? Any fusion step? Which one? Please make this point clear in the caption of that figure."
Answer: *For Learning to Rank, we adopt ANTs SyN as the registration method and Joint Label Fusion as the fusion method. We have added explanations both in the caption and implementation details.*

Please refer to ***Line 566-568, Figure 6*** *in the manuscript for a detailed description.*

Question 21: "Fig. 6. There is an artifact (false contour which possible saturation of the intensity) for the LPBA40 and Elastix case images. Please fix or explain the reason for that."
Answer: *This is because of the preprocessing steps in Elastix and visualization adjustment in ITK-SNAP. We have added explanations on the visualization of these false contours.*

Please refer to ***Line 650-652*** *in the manuscript for a detailed description.*

Question 22: "Concerning results of fig. 9, the authors said that there is a linear relationship between the two considered variables. Two is fine for the second and third rows. However, for the proposed method (row 1), I do not see a clear linear relationship. In fact, the linear regression does not explain much of the cloud of points. Please, take this fact into account and modify your

conclusions accordingly."
Answer: *We have rephrased the discussion so that the linear relationship conclusion does not hold for the results with our method but it should hold for the expected Dice.*

*Please refer to **Line 680-683, 691-693** in the manuscript for a detailed description.*

Question 23: "In experiment concerning fig. 9, the Dice coefficient is represented as a function of the corresponding amount of deformation. However, the atlas selection is done using the final energy of registration part (using the MRF formulation). Why not to compare the Dice to the energy or deformation to energy, for instance? This will justify the part given in lines 666- (page 29). For instance, a figure similar fig. 10, but showing the energy and atlas number after the sorting."
Answer: *We adopt the deformation amount in consideration of a fair and consistent comparison among the three methods because they do not share the same energy function. However, the deformation amount can be computed from the deformation field outputs for all of the three registration methods.*

*Please refer to **Line 676-679** in the manuscript for a detailed description.*

Question 24: "A section showing the "Disk Space Usage" is presented. However, I think the "memory need" for the algorithms to run is nowadays even more important than the "disk usage". Is possible to add a table similar to table 5, with the memory used? Add a paragraph with a discussion about the achieved results in the main text. Some mention is given in the discussion section (line 768), but in my opinion, a table should be included with memory usage and done for the disk."
Answer: *We have added memory usage results in the discussion section.*

*Please refer to **Line 824-831, Table 8** in the manuscript for a detailed description.*

Question 25: "Concerning the meta-parameters of the proposed algorithm (t, eta, alpha, etc.), in lines 557- (page 23) some explanation and values used are provided. Please add a paragraph in the "Discussion" about this point: how to select these parameters for a given scenario, how sensitive will the results be with respect to the selection of these meta- parameters, and so forth."
Answer: *We have added one paragraph providing guidance on how to choose the hyper-parameters in addition to evaluation criteria with cross-validation.*

*Please refer to **Line 764-773** in the manuscript for a detailed description.*

Question 26: "Last paragraph in the Discussion deals with Deep Learning (DL) and Artificial Intelligence (AI). However, I see three places DL/AI algorithms can be applied in the context of the paper: in the registration part, in the atlas selection part and in the fusion part. DL/IA can also be applied as a whole: as a pure segmentation algorithm. In my opinion, the authors should elaborate more about that point."

Answer: *We have revised the statement such that deep learning approaches can be incorporated into our framework in any of the components or replace it as a whole.*

*Please refer to **Line 881-883, 891-893** in the manuscript for a detailed description.*

Question 27: "In lines 849-850, the manuscript reads: "The results demonstrate that our method achieves comparable performance compared to some competitive brain segmentation tools in terms of computation time, accuracy, scalability and disk usage.", I cannot see evidence of that fact. Authors compare their algorithm with respect to other atlas-based segmentation algorithms. However, no comparison is performed with respect to other segmentation algorithms in general. Paper can conclude that the proposed method outperforms other atlas-based segmentation found in the literature (in execution time, accuracy, etc.). Please justify or change that statement."
Answer: *Thanks for pointing it out. We have changed the statement so that we conclude that we outperform some atlas-based method in the literature.*

*Please refer to **Line 903-904** in the manuscript for a detailed description.*

Question 28: "I do not see the need to include ref. 105: in my opinion, it is an out-of-scope work: diabetes has nothing to do with the content of the paper."
Answer: *We agree that this reference has almost nothing to do with this work. We added this reference according to a suggestion of Reviewr #2, who has not responded to our last rebuttal. Anyway, we have removed this reference.*

References

[1] Ce Liu, Jenny Yuen, and Antonio Torralba. Nonparametric scene parsing via label transfer. *IEEE Transactions on Pattern Analysis and Machine Intelligence*, 33(12):2368–2382, 2011.

[2] Abdel Aziz Taha and Allan Hanbury. Metrics for evaluating 3d medical image segmentation: analysis, selection, and tool. *BMC medical imaging*, 15(1):1–28, 2015.

[3] Abhijit Guha Roy, Sailesh Conjeti, Nassir Navab, Christian Wachinger, Alzheimer's Disease Neuroimaging Initiative, et al. Quicknat: A fully convolutional network for quick and accurate segmentation of neuroanatomy. *NeuroImage*, 186:713–727, 2019.
[3]
