# Supplementary material for: Integrated 3d flow-based multi-atlas brain structure segmentation
Source: PLoS One. 2022 Aug 15;17(8):e0270339. doi: 10.1371/journal.pone.0270339 (PMC9377636; doi:10.1371/journal.pone.0270339)
Supplement: Attachment — Submitted filename: Response to Reviewers.pdf [file pone.0270339.s003.pdf]

Integrated 3d flow-based multi-atlas brain structure segmentation

Authors' Response

Yeshu Li Ziming Qiu Xingyu Fan Xianglong Liu
Eric I-Chao Chang Yan Xu

We thank the editor and reviewers for the constructive comments and the general appreciation of our work. We have revised our manuscript (manuscript number: PONE-D-21-07784R2) accordingly. Please see below our point-by-point responses to the specific questions.

1 Response to Reviewer #1's Comments

Question 1: "For the question of the smoothness, the author response that the discontinuity is actually desirable in applications like brain image registration, while the reviewer has a different view on this. Most latest brain registration can theoretically preserve the smoothness (like Demons, ANTs, Voxel Morph etc.). Although practically, some discontinuity may rise due to the discretization. The smoothness is also important for accurate alignment, especially when the deformation is large. While the proposed method seems can not preserve the smoothness in theory. In Fig. 4, the author visualized the deformation field with stronger smoothness generated with integrated flow. So is that means in practical, the proposed method can generate the smooth deformation field in most cases?"
Answer: *Thanks for your comment. Our response to the related question in the previous revision may be confusing. We have revised the statements on how our method controls smoothness of the produced deformation fields. Global smoothness is actually preserved by our method because the differences between the displacement vectors of adjacent vectors are minimized jointly. The weighted truncated norm further controls local smoothness with smoother fields produced by larger* α *and* d*. We agree that smoothness is important for accurate alignment. Although the integrated flow yields a discrete point-to-point correspondence, which may not look smooth intuitively, the registration result takes into account the three terms in MRFs, and usually gives point correspondences translated to an image as smooth as possible. Our method provides a way of adjusting*

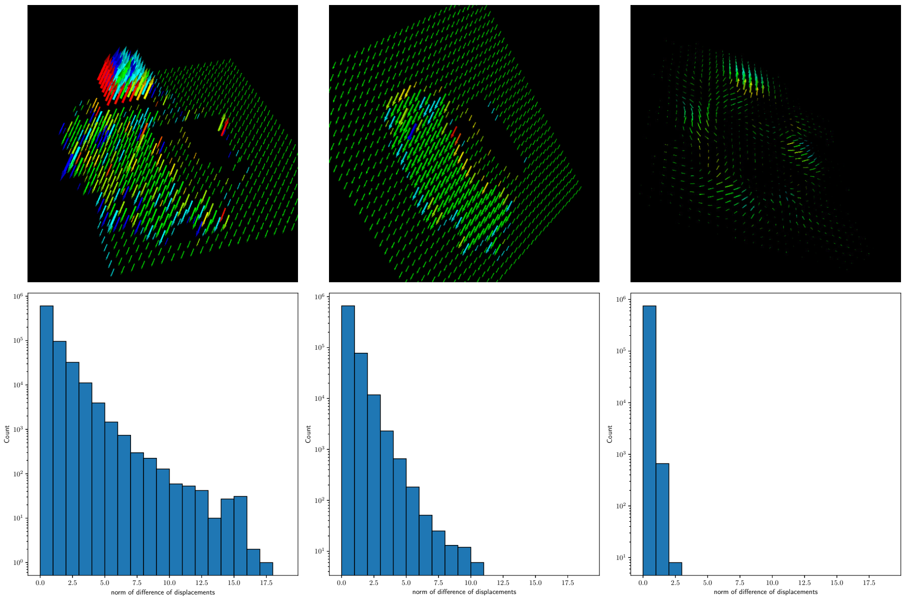

Figure 4: **Examples of the visualized displacement fields and their corresponding histogram statistics. The histograms are based on the smoothness terms for all pairs of adjacent voxels with $\alpha = 1$ and $d = \infty$. From left to right: the integrated flow with less restriction on local smoothness; the integrated flow with stronger local smoothness; ANTs SyN.**

trade-offs among feature matching, conservative fields and smooth fields. Furthermore, smoothing is applied during feature extraction.

*Please refer to **Line 252-269, Figure 4** in the manuscript for a detailed description:*
"In this energy function, truncated L1 norms are adopted in both the data term and smoothness term to combat outliers and preserve flow discontinuities respectively. Intuitively, if the L1 norm in the smoothness term is not truncated, the final flow field will be smooth locally and globally. In general, the registration/mapping field observes regularity in the global smoothness, while encountering local discontinuity at the boundary of the anatomical structures. On one hand, our proposed method consists of a regularization term that encourages the smoothness of the field; on the other hand, the SIFT features capture the important anatomical structures that observe robustness and informativeness to be able to model local discontinuity in the registration map. The truncated norms thus control the level of allowed local discontinuity while global smoothness is always imposed with $\alpha > 0$. We give an example of the displacement fields produced by our method and ANTs in Fig. 4 with weak and strong local smoothness. The histograms are based on the smoothness terms for all pairs of adjacent vox-

els with $\alpha = 1$ and $d = \infty$. Note that our method produces less smooth fields than ANTs because of the discrete nature of integrated flows. However, smoother displacement fields can be obtained by choosing larger α and d (the middle column in Fig. 4). The displacement fields of our method are smooth overall since 90% *of the gradients of displacements are under* 2.0 *(the left column in Fig. 4).”*

2 Response to Reviewer #3’s Comments

We thank the reviewer for the useful comments. All the co-authors have proof-read the whole paper once more and fixed the found typos.

References
